# Epigenome editing strategies for the functional annotation of CTCF insulators

**DOI:** 10.1038/s41467-019-12166-w

**Published:** 2019-09-18

**Authors:** Daniel R. Tarjan, William A. Flavahan, Bradley E. Bernstein

**Affiliations:** 1 Department of Pathology and Center for Cancer Research, Massachusetts General Hospital and Harvard Medical School, Boston, MA 02114 USA; 2 Broad Institute of Harvard and MIT, Cambridge, MA 02142 USA

**Keywords:** Genetic engineering, Gene regulation, Chromatin, Chromatin structure, CRISPR-Cas9 genome editing

## Abstract

The human genome is folded into regulatory units termed ‘topologically-associated domains’ (TADs). Genome-wide studies support a global role for the insulator protein CTCF in mediating chromosomal looping and the topological constraint of TAD boundaries. However, the impact of individual insulators on enhancer-gene interactions and transcription remains poorly understood. Here, we investigate epigenome editing strategies for perturbing individual CTCF insulators and evaluating consequent effects on genome topology and transcription. We show that fusions of catalytically-inactive Cas9 (dCas9) to transcriptional repressors (dCas9-KRAB) and DNA methyltransferases (dCas9-DNMT3A, dCas9-DNMT3A3L) can selectively displace CTCF from specific insulators, but only when precisely targeted to the cognate motif. We further demonstrate that stable, partially-heritable insulator disruption can be achieved through combinatorial hit-and-run epigenome editing. Finally, we apply these strategies to simulate an insulator loss mechanism implicated in brain tumorigenesis. Our study provides strategies for stably modifying genome organization and gene activity without altering the underlying DNA sequence.

## Abstract (web summary)

The role of CTCF-bound insulator elements in enhancer-gene interactions and transcriptional regulation remains poorly understood. Here, the authors investigate multiple epigenome editing strategies for perturbing individual CTCF-bound insulators, and evaluate their effects on genome topology and transcription.

## Introduction

TAD structure is conserved across mammalian cell types and tissues^1,2^, and forms the foundational structure within which cell type-specific enhancers control cell state^3,4^. Indeed, the majority of gene-enhancer interactions appear to occur within TADS. Within these conserved topological structures, dynamic interactions between enhancers and promoters modulate lineage-specific gene expression. The DNA binding insulator protein CTCF plays important roles in controlling enhancer-promoter interactions, and directing TAD boundary formation^4–8^. In certain cancers, CTCF motifs are frequently compromised by DNA mutations or methylation events that prevent binding^9,10^. Despite broad relevance to development and disease, CTCF insulators and TAD boundaries remain poorly understood.

The complexity of genome topology, gene-enhancer connectivity and cell type-specific enhancer activity suggests that the functional significance of individual CTCF-bound insulators are likely to be highly context-specific^5,6,11,12^. In particular, the consequence of CTCF loss in a given cell type will depend on the ensuing topological alteration, as well as the state of nearby genes, enhancers and sequence elements. Adding to this complexity, TAD boundaries often contain multiple CTCF binding sites^1^. Accordingly, we need better strategies to test the context-specific functions of individual CTCF sites, and ultimately to predict the consequences of disease-associated insulator disruptions. While genome and epigenome editing tools have been used to disrupt a small number of CTCF sites^9,13^, generalizable strategies for specific, and stable (mitotically heritable) epigenetic disruption of insulators are needed.

**Fig. 1:**
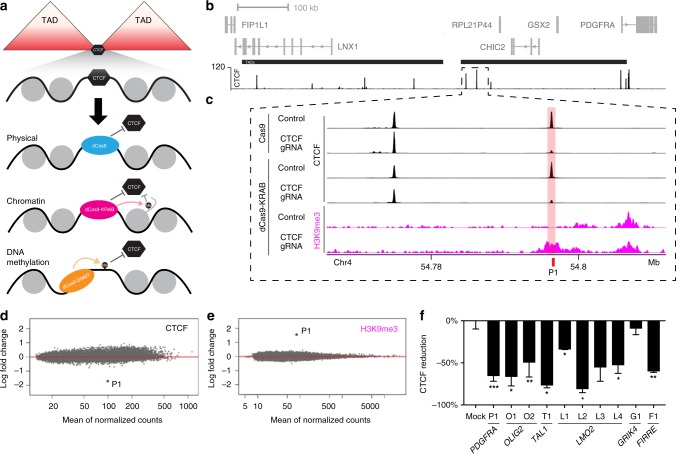
Epigenome editors can specifically disrupt CTCF binding at topological insulators. **a** Schematic depicts potential epigenome editing strategies for displacing CTCF from a theoretical insulator separating two TADs. **b** Genomic view of the PDGFRA locus on chromosome 4 shows genes (gray), two TADs (black bars, middle) and CTCF ChIP-seq signal for HEK293 cells (black, bottom). **c** Expanded view of the boundary region flanking the TAD that contains the PDGFRA promoter shows ChIP-seq signals for CTCF (black) and H3K9me3 (pink). CTCF profiles are shown for HEK293 cells after epigenome editing by Cas9 or dCas9-KRAB, with gRNA to the PDGFRA insulator P1 CTCF target site (pink shade) or a non-targeting control. H3K9me3 profiles are shown for HEK293 cells after epigenome editing by dCas9-KRAB, with gRNA to the PDGFRA insulator P1 CTCF target site or a non-targeting control. **d**, **e** Plots show differential ChIP-seq signals for CTCF (**d**) or H3K9me3 (**e**) over all CTCF peaks genome-wide, in cells expressing dCas9-KRAB with P1 targeting gRNA (relative to control). Each point represents the log fold change in normalized read counts observed at that locus, ordered by the mean count observed across all conditions. CTCF occupancy is reduced and H3K9me3 is increased specifically over the targeted P1 CTCF site. **f** Bar plots show change in CTCF occupancy measured by ChIP-qPCR over indicated CTCF sites following transient transfection with dCas9-KRAB and indicated gRNA (see also Fig S1). CTCF disruption by epigenome editing is robust across the ten individually targeted loci. Data are normalized to non-targeting controls. Error bars, mean ± s.e.m. *P < 0.05, **P < 0.01, ***P < 0.001 by Student’s *t*-test. *n* = 4 (P1 and O1) or 2 (other loci) biological replicates, respectively. Source data for 1f are provided as a Source Data file, ChIP-seq and RNA-seq data can be found via GEO accession GSE121998

Here, we systematically test different combinations of CRISPR-based epigenome editors for their ability to disrupt individual topological insulators in their endogenous context, with specificity and stability, and without the limitations associated with DNA editing^14^ (Fig. 1a). We show that fusions of dCas9 to transcriptional repressors (dCas9-KRAB) and DNA methyltransferases (dCas9-DNMT3A, dCas9-DNMT3A3L) can be used to selectively displace CTCF from specific insulators, but only when precisely targeted to the cognate motif. We further demonstrate that stable, partially heritable insulator disruption can be achieved through combinatorial hit-and-run epigenome editing with dCas9-KRAB and dCas9-DNMT3A3L. Finally, we apply these strategies to activate *PDGFRA* expression in glioblastoma stem cells, thus simulating an insulator loss mechanism implicated in brain tumorigenesis.

## Results

### Epigenome editing can disrupt CTCF at topological insulators

**Fig. 2:**
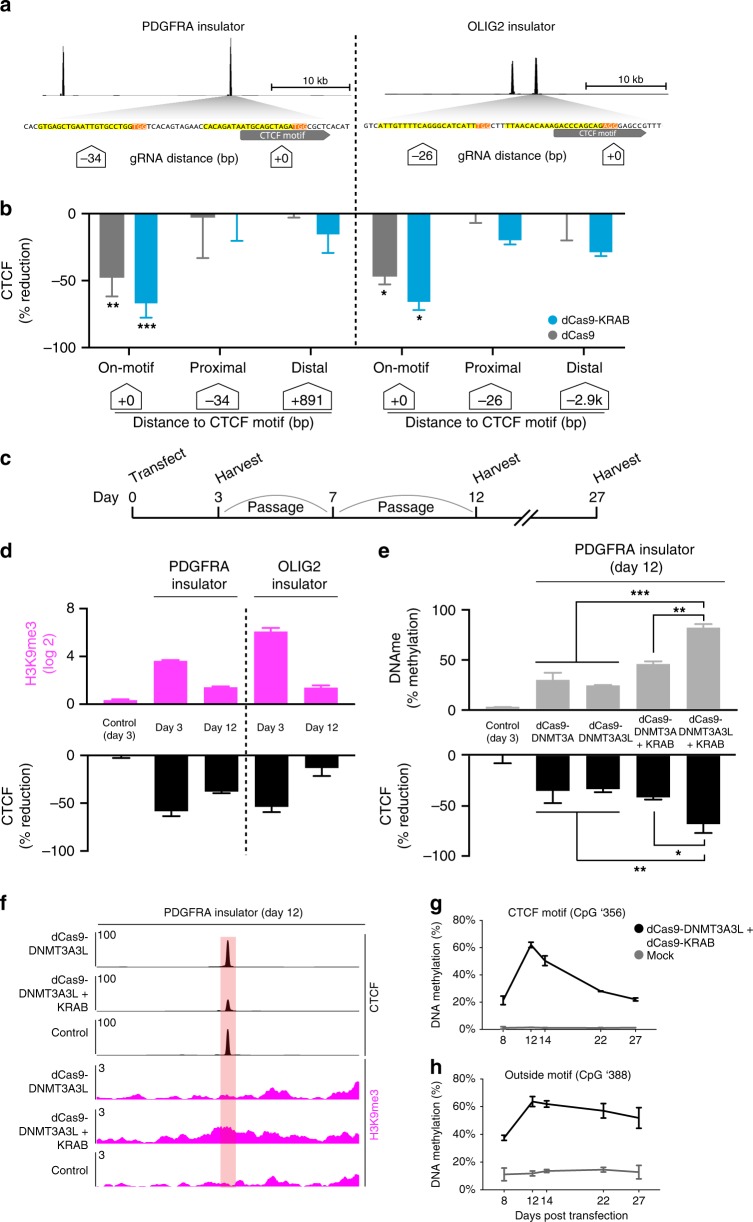
Locus-specific DNA methylation confers stable CTCF disruption. **a** Schematic depicts CTCF sites in the PDGFRA and OLIG2 insulators. CTCF ChIP-seq signal is shown for HEK293 cells (top, black). Expanded view below shows underlying DNA sequence. gRNAs were designed to directly target the CTCF motifs or proximal sequence. CTCF motifs (gray), gRNA PAMs (orange) and gRNA spacers (yellow) are highlighted. White boxes indicate distance of each gRNA to CTCF motif. **b** Bar plots show change in CTCF occupancy at the PDGFRA (left) or OLIG2 (right) insulators in HEK293 cells transfected with indicated epigenome editing construct and gRNA, as measured by ChIP-qPCR. Only gRNAs targeted directly to the CTCF motif significantly reduced CTCF binding. White boxes indicate distance of each gRNA to CTCF motif. Error bars, mean ± s.e.m. *n* ≥ 3. *P < 0.05, **P < 0.01, ***P < 0.001 by Student’s *t*-test. (See also Fig S2). **c** Schematic depicts the experimental time course in panels **d**–**g**. Cells were transiently transfected with epigenome editing reagents plus either a single targeting gRNA or a non-targeting control, and passaged as indicated until expression of the construct was no longer detectable (see also Fig. S2F). **d** Bar plots show change in H3K9me3 (top, pink) or CTCF occupancy (bottom, black) measured by ChIP-qPCR at the PDGFRA or OLIG2 insulators at days 3 and 12 post transfection with dCas9-KRAB plus single targeting gRNAs or a non-targeting control. CTCF occupancy normalized to mock transfected controls. Error bars, mean ± s.e.m. *n* ≥ 2. **e** Bar plots show change in DNA methylation measured by methylation sensitive restriction followed by qPCR (top/gray), and change in CTCF occupancy measured by ChIP-qPCR (bottom, black), at the P1 CTCF site in the PDGFRA insulator at 12 days post transfection with the indicated combination of epigenome editing reagents. CTCF reduction normalized to mock transfected controls. Data analyzed by two-way ANOVA. Error bars are mean ± s.e.m. *n* ≥ 2. **f** ChIP-seq tracks for CTCF (black) and H3K9me3 (pink) at the P1 insulator site at 12 days post transfection with the indicated epigenome editing reagents, plus a single gRNA targeted to the P1 site or a non-targeting control. **g**–**h** Plots show changes in DNA methylation of two CpGs in the PDGFRA P1 site following transient transfection with dCas9-DNMT3A3L + dCas9-KRAB plus a single gRNA targeted upstream of the CTCF motif (See also Supplementary Table 1). Cells were harvested at the days indicated (*x*-axis). DNA methylation was assessed by bisulfite sequencing. Error bars are mean ± s.e.m. *n* = 2. Insulator methylation is maintained in dividing cells after initiation by transient epigenome editing reagents. Source data for 2b, 2d, 2e, 2g are provided as a Source Data file. ChIP-seq and RNA-seq data can be found via GEO accession GSE121998

Fusions of catalytically dead Cas9 (dCas9) to the Krüppel-associated box (KRAB) repressor (dCas9-KRAB) can be targeted to specific loci by gRNAs, where they catalyze histone H3 lysine 9 methylation (H3K9me3) and can repress target gene expression and enhancer function^12,15–20^. We therefore tested the capacity of dCas9-KRAB to displace CTCF from its binding motif. We initially focused on CTCF-bound insulators that demarcate a TAD boundary upstream of a known glioma oncogene, *PDGFRA* (Fig. 1b). This locus shows the hallmarks of a TAD boundary by Hi–C and contains two CTCF sites ~20 kb apart, both of which are strongly bound in HEK293 cells (Fig. 1b, Supplementary Fig. 1A). We designed a guide RNA (gRNA) targeting the CTCF motif closer to the TAD interior (annotated as site P1 in Fig. 1c), and also incorporated 8 bases of proximal genomic sequence to ensure specificity (Fig. 2a). We expressed dCas9-KRAB and the CTCF targeting gRNA in HEK293 cells by lentiviral transduction and mapped CTCF binding and H3K9me3 enrichment by genome-wide chromatin immunoprecipitation and sequencing (ChIP-seq). Targeting dCas9-KRAB to this single CTCF site achieved an 83% reduction in CTCF binding, with concomitant enrichment of H3K9me3 across a 3 kb region around the targeted site (Fig. 1c, Supplementary Fig. 1G). The observed 3 kb spreading of the histone modification is consistent with previous studies that have localized dCas9-KRAB to other regulatory elements (Supplementary Fig. 1I, 1J)^15^. Importantly, CTCF binding at the non-targeted proximal CTCF site within the TAD boundary region was unchanged (Supplementary Fig. 1E).

We next benchmarked dCas9-KRAB-mediated disruption of CTCF binding against Cas9-mediated genome editing of the underlying motif sequence. We targeted Cas9 to the P1 CTCF site in the *PDGFRA* TAD boundary using the same gRNA as the experiments above. The reduction in CTCF binding at the Cas9 genome edited CTCF site was comparable to the dCas9-KRAB epigenome edited cells (81% vs. 83%, respectively) (Fig. 1c, Supplementary Fig. 1F). We conclude that epigenome editing using dCas9-KRAB can disrupt CTCF binding with similar efficacy to genome editing.

To evaluate the specificity of epigenome editing systematically, we used ChIP-seq to map H3K9me3 and CTCF genome-wide in HEK293 cells after CTCF disruption by dCas9-KRAB (Fig. 1d, e). The P1 CTCF site (on-target) showed significant reduction in CTCF binding compared to non-targeted controls. We also observed concomitant H3K9me3 enrichment at the target site. Remarkably, we did not detect significant alteration at any of the other ~60,000 CTCF binding sites. These data demonstrate that this epigenome editing strategy can effectively disrupt this CTCF insulator with exquisite specificity.

We next expanded our study to test dCas9-KRAB-mediated disruption of CTCF binding at ten individual CTCF binding sites. This set included TAD boundaries known to insulate the oncogenes *OLIG2*, *TAL1*, and *LMO2*, in addition to *PDGFRA*^9,10^. As with the *PDGFRA*-proximal TAD boundary, the selected loci also show features of TAD boundaries by Hi–C and CTCF binding (Supplementary Fig.  1B-D). In order to target a biologically representative set, we selected TAD boundaries with between one and three CTCF binding sites (Fig. 1f, Supplementary Fig.  1G, 1H). We transiently expressed dCas9-KRAB in separate experiments with gRNAs targeting 10 different CTCF motifs. These experiments succeeded in significantly reducing CTCF binding at 8 out of 10 sites tested (Fig. 1f). We also tested integrated expression constructs encoding dCas9-KRAB and gRNA targeting a subset of these loci. These stable expression constructs reduced CTCF binding at all tested loci, including the GIRK4 proximal TAD boundary that was refractory to the transient expression strategy (Supplementary Fig.  1H). These collective results demonstrate that the epigenome editor dCas9-KRAB can selectively disrupt individual CTCF binding sites, despite the high genomic prevalence of the binding motif.

### Locus-specific DNA methylation confers stable disruption

We next considered the mechanistic basis of CTCF displacement by dCas9-KRAB. We specifically sought to evaluate the relative contributions of binding site occlusion by dCas9 versus the repressive chromatin state mediated by the KRAB domain. To decouple these effects, we targeted dCas9 lacking the KRAB domain to CTCF motifs in two of the TAD boundaries evaluated above. We found that transient expression of dCas9 alone with gRNA reduced CTCF binding at both sites, although to a lesser degree than the fusion (Fig. 2a, b). We also tested whether CTCF displacement required gRNAs that directly target the CTCF motif, or whether this could also be achieved by targeting nearby sequences. We designed gRNAs to target dCas9-KRAB to sequences adjacent to CTCF motifs in the *PDGFRA* and *OLIG2* TAD boundaries (Fig. 2a, b). Although these experiments increased H3K9me3 levels at the target loci (Supplementary Fig. 2A), their impact on CTCF binding was minimal (Fig. 2b). These data suggest that this epigenome editing fusion acts primarily through binding site occlusion and secondarily through chromatin repression to displace CTCF from TAD boundaries.

A distinguishing feature of epigenome editing is its potential to induce changes that are transient or, alternatively, are stably maintained through cell division. We therefore investigated the temporal stability of H3K9me3 enrichment and CTCF depletion incurred by dCas9-KRAB. We transiently expressed dCas9-KRAB targeted to the *PDGFRA* or *OLIG2* insulators in HEK293 cells. We then serially passaged the cells for 12 days, at which point dCas9-KRAB protein could not be detected (Fig. 2c). At early time points, H3K9me3 enrichment and reduced CTCF binding were readily detected (Fig. 2d). However, as dCas9-KRAB expression was lost, histone methylation was also lost and CTCF binding largely restored to control levels (Fig. 2d). Our results indicate that CTCF displacement requires continuous dCas9-KRAB expression, as is consistent with prior studies that have used this fusion to repress chromatin and enhancer activity^12,15–19^.

We therefore explored whether other epigenome editing strategies could confer stable, mitotically heritable (or at least partially heritable) displacement of CTCF insulators. Fusions of dCas9 to DNA methyltransferase 3A (dCas9-DNMT3A) and optimized derivatives (e.g., dCas9-DNMT3A3L) have been used to direct DNA methylation to specific loci and silence promoters, with varying degrees of stability^13,21–25^. CTCF binding is sensitive to DNA methylation status^7,8^, and can be disrupted by targeted dCas9-DNMT3A^13^. We tested the ability of dCas9-DNMT3A and dCas9-DNMT3A3L to disrupt CTCF binding at the *PDGFRA* insulator in HEK293 cells. We transiently expressed dCas9-DNMT3A or dCas9-DNMT3A3L for 3 days, and measured DNA methylation by bisulfite sequencing. We detected methylation over the targeted CTCF motif on 20 to 40% of alleles, with DNMT3A3L more effective than DNMT3A (Supplementary Fig. 2B). Importantly, DNA methylation over the CTCF motif persisted after the epigenome edited cells were serially passaged. We observed ~20% DNA methylation and a congruent ~20% reduction in CTCF binding 12 days after the transient transfection, even though the dCas9 fusions were no longer detected (Fig. 2e, Supplementary Fig. 2F).

Although the DNA methyltransferase fusions induce more stable changes, they were relatively less effective at displacing CTCF. We therefore tested whether the combination of dCas9-targeted H3K9me3 and DNA methylation, used previously for ‘hit-and-run’ epigenetic gene silencing^26,27^, could disrupt CTCF insulators with greater efficacy and stability. We transiently co-expressed dCas9-KRAB with either dCas9-DNMT3A or dCas9-DNMT3A3L, along with gRNA targeting the CTCF motif in the *PDGFRA* insulator. We found in particular that the dCas9-KRAB/dCas9-DNMT3A3L combination markedly increased DNA methylation and reduced CTCF binding. Remarkably, robust CTCF disruption was still evident 12 days after transient expression of this fusion, despite lack of continued protein expression (Fig. 2e, f, Supplementary Fig. 2F). To confirm that the altered insulator state induced by dCas9-KRAB/dCas9-DNMT3A3L is epigenetically maintained through mitosis, we continued the time course out to 27 days (Fig. 2g, h, Supplementary Fig. 2C). Methylation at the target locus peaked at 12 days, but was still maintained at 27 days (Fig. 2g, h). At this extended time point, the CpG within the CTCF motif (CpG ‘356) retained >20% DNA methylation while a nearby CpG (CpG ‘388, 32 bases from the motif) retained >50% methylation, near its peak level of ~64% (Fig. 2g, h). This rate of decline is considerably slower than expected from passive demethylation associated with cell divisions (Supplementary Fig. 2D), supporting that the DNA methylated state is epigenetically maintained through mitosis. To evaluate specificity of editing, we performed ChIP-seq on HEK293 cells 12 days after transient transfection with the dCas9-KRAB/dCas9-DNMT3A3L combination. This confirmed robust H3K9me3 enrichment and CTCF depletion at the target insulator, with few significant alterations at other CTCF binding sites in the genome (Fig. 2f, Supplementary Fig. 2E). We conclude that the dCas9-KRAB/dCas9-DNMT3A3L combination can potently disrupt CTCF insulators and initiate a DNA methylated state that is stably maintained after withdrawal of the epigenome editors.

### Dissecting topological mechanisms of gene control

**Fig. 3:**
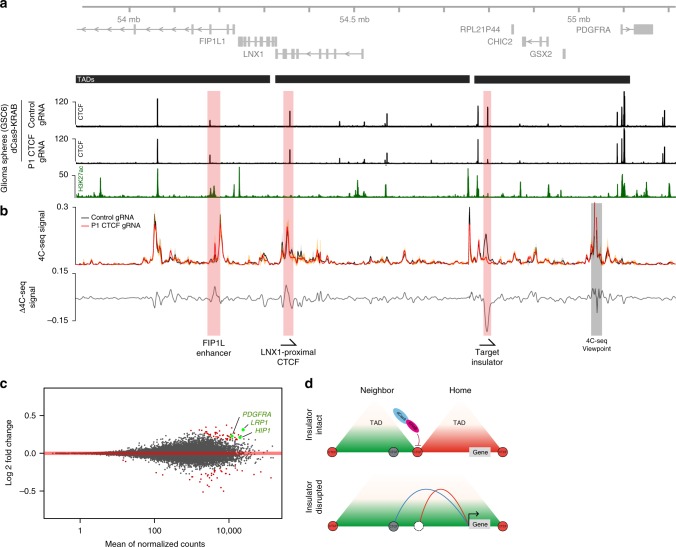
Dissecting topological mechanisms of gene control with epigenome editors. **a** Genomic view of the expanded PDGFRA locus on chromosome 4 shows genes in gray (top). The region shown is divided into three TADs (black bars, middle), per HiC data. ChIP-seq signal tracks for CTCF (black) are shown for GSC6 cells following transfection with dCas9-KRAB plus single gRNA targeting the P1 CTCF site or non-targeting control. ChIP-seq for H3K27ac (a marker of regulatory activity) in GSC6 is shown green. **b** Plot shows 4C-seq signal measured from a viewpoint near the PDGFRA promoter. GSC6 cells were transfected with gRNA targeting the P1 CTCF site (red) or non-targeting control (black), as in **a**. Signal is shown as the mean across replicates (solid line) ± one standard deviation (shaded area around mean). The difference in 4C-seq signal between the two conditions is shown below the 4C-seq signal (gray line, bottom). Epigenome editing of the P1 site with dCas9-KRAB decreases interactions between the PDGFRA promoter and the CTCF insulator, and modestly increases interactions between the PDGFRA promoter and sites in the adjacent TAD, including the LNX1 proximal CTCF site, and the previously described FIP1L1-proximal enhancer. **c** Plot shows differential gene expression measured by RNA-seq from GSC6 cells following epigenome editing with dCas9-KRAB and single gRNA targeted to the P1 site or a non-targeting control. Differentially expressed genes (*P*< 0.05) are highlighted (red). Differential PDGFR pathway genes are indicated (green). **d** Schematic depicts consequences of insulator disruption by epigenome editing. In glioma cells, insulator disruption at the PDGFRA locus increases interactions across the disrupted insulator, which may allow enhancers to contact sites in the neighboring TAD. Data are available via GEO accession GSE121998

We next sought to use this epigenome editing strategy to model a disease-associated insulator loss event. We previously reported that disruption of an insulator in the *PDGFRA* locus by aberrant DNA methylation is associated with increased expression of the oncogene in IDH-mutant gliomas. We showed that disruption of the CTCF motif by genome editing increased *PDGFRA* expression in patient-derived gliomasphere models^9^. This prompted us to explore whether epigenome editing could alter *PDGFRA* locus topology and expression without changing underlying DNA sequence. We used the GSC6 gliomasphere model for this purpose, which retains an intact *PDGFRA* insulator and expresses the oncogene at low levels. We used lentiviral transduction to achieve consistent expression of dCas9-KRAB and gRNA in GSC6 cells. We then mapped CTCF, H3K9me3, and the enhancer-associated marker H3K27 acetylation (H3K27ac) by ChIP-seq. We detected a 95% reduction in CTCF binding at the *PDGFRA* insulator, while adjacent CTCF sites were unchanged (Fig. 3a, Supplementary Fig. 3A, 3B).

In IDH-mutant gliomas, *PDGFRA* insulator loss is associated with an altered locus topology that allows the *PDGFRA* promoter to interact with an enhancer in an adjacent TAD^9^. We therefore investigated how *PDGFRA* promoter interactions were altered by epigenome editing, using 4C-seq. We found that CTCF displacement by epigenome editing markedly reduced the interaction between the promoter and insulator, which reside at opposite sides of the TAD, consistent with loss of a long-range chromosomal loop that normally connects these loci (Fig. 3b). Displacement of the insulator also caused a qualitative increase in long-range interactions between the PGDFRA promoter and several sites in the adjacent TADs. These changes were less striking than the loss of interactions with the target insulator, but were also consistent with TAD boundary loss. Putative sites of interaction include the LNX1 proximal CTCF site, and the previously described FIP1L1-proximal enhancer^9^ (Fig. 3a, b, Supplementary Fig. 3C, 3D). We conclude that epigenome editing can disrupt this TAD boundary, alter locus topology and allow aberrant cross-boundary enhancer-promoter interactions.

We next investigated how *PDGFRA* insulator disruption altered gene expression in GSC6 cells using RNA-seq. Insulator disruption increased expression of *PDGFRA* and several downstream PDGFR pathway genes (Fig. 3c). We did not detect significant changes in the expression of other genes in the *PDGFRA* TAD or the adjacent TAD, although several genes in a more distal upstream TAD were modestly induced (Supplementary Fig. 3E). Overall, *PDGFRA* scored as the most significant differentially expressed gene in this locus (Supplementary Fig. 3F).

Finally, we investigated how insulator disruption might alter intra-TAD interactions of an inactive promoter. We designed a 4C viewpoint to the promoter of *GSX2*, which is neither expressed in GSC6 gliomaspheres nor activated by insulator disruption (Supplementary Fig. 4A, 4B). We found that the interaction between the GSX2 promoter and the *PDGFRA* insulator was reduced by disruption of the latter, albeit to a lesser extent than that observed for the *PDGFRA* promoter viewpoint. We found that insulator disruption also impacted long-range interactions made by the *GSX2* promoter, particularly with H3K27ac-marked sites in the adjacent TAD. This suggests that CTCF displacement by epigenome editing alters the overall folding of this region, with context-specific and promoter-specific effects. Taken together, our results demonstrate the potential of epigenome editing to model insulator loss events and evaluate consequential changes in genome topology and gene expression (Fig. 3d).

## Discussion

In summary, we present epigenome editing strategies for potent, specific, and stable disruption of CTCF insulators (Fig. 1a). We demonstrate the applicability of a combinatorial ‘hit-and-run’ strategy to robustly disrupt insulators and establish a DNA methylated state that is epigenetically maintained after withdrawal of the epigenome editors. Alternate strategies may be employed to achieve either transient or stable insulator disruption. Another advantageous feature of epigenome editing, relative to genome editing, is the remarkable specificity with which an insulator can be disrupted, as evidenced by lack of detectable off-target effects in genome-wide analyses. Our data also provide mechanistic insight into an insulator loss event associated with oncogene activation by simulating the disease-associated lesion without altering the underlying genetic sequence. Our data faithfully simulate a cancer-associated insulator loss and confirm that it allows the *PDGFRA* oncogene to engage in multiple long-range interactions with a neighboring TAD, including with a super-enhancer element that likely drives its expression (Fig. 3d). Looking forward, these strategies can provide powerful and general means for dissecting the roles of insulators in endogenous contexts, for simulating and evaluating causality of disease associated lesions, and for precisely controlled modulation of genome topology in experimental, and ultimately, therapeutic settings.

## Methods

### Cell culture and transfection

HEK293 culture and passaging: The human embryonic kidney cell line HEK293 (ATCC, CRL-1573) was cultured in DMEM with 10% FBS, 1% Pen/Strep antibiotic mix (Gibco).

HEK293 transient transfection: For transfections, 10 cm plates were seeded 48 h in advance with 2 × 10^6^ cells per plate. To make transfection mix 8 µg of dCas9 effector plasmid DNA and 1 µg of guide RNA expression plasmid were re-suspended in Opti-MEM media (Thermo). For combinations of effectors, total transfected DNA was kept constant at 8 µg. Fugene HD (Promega) was added to transfection mix according to the manufacturer protocol using a 3:1 ratio to DNA. Cells were harvested 72 h after transfection for ChIP, DNA, or RNA extraction.

HEK293 serial passaging: For serial passaging experiments, 90% of cells were harvested and remaining cells were passaged into fresh media in a new 10 cm dish. Cells were passaged every 72 h until final harvest.

GSC6 culture and passaging: GSC6 gliomasphere lines were derived from *IDH* wild-type tumors resected at Massachusetts General Hospital^28^. These cell lines were established under Protocol # 2005P001609, which was approved by The Partners Human Research Committee (PHRC), and complies with all relevant ethical regulations. Gliomaspheres were maintained in culture as described^9,29,30^. Neurosphere cultures contain Neurobasal media supplemented with 20 ng ml^−1^ recombinant EGF (R and D Systems), 20 ng ml^−1^ FGF2 (R and D Systems), 1 × B27 supplement (Invitrogen), 0.5 × N2 supplement (Invitrogen), 3 mM L-glutamine, and penicillin/streptomycin. Cultures were confirmed to be mycoplasma-free via PCR methods.

### Plasmids

Plasmids were purchased from Addgene including dCas9-KRAB, dCas9-KRAB-T2a-Puro, pdCas9-DNMT3A-EGFP (Catalog numbers 50919, 71236, 71666, respectively). Expression vector for dCas9 (JDS286) was a gift from J. Keith Joung. Human codon optimized sequence for dCas9–Dnmt3a3l^24^ was synthesized (Genscript) and cloned into pcDNA3.1 including an in-frame P2A-eGFP fusion. All plasmids are available from Addgene: pcDNA3.1-dCas9-Dnmt3a-Dnmt3l-P2A-eGFP: ID 128424. Expression vectors including dCas9-Dnmt3a-Dnmt3l and the gRNA expression cassette are also available from Addgene: pDT304 (PuroR): ID 128800, and pDT305 (eGFP): ID 128801. SpCas9 and guide RNA expression vectors lentiCRISPR and lentiGuide were gifts from the Broad Genomic Perturbation Platform.

### Guide RNA target selection and expression vector cloning

Insulator loci were identified in previous studies and by selecting CTCF binding sites near conserved Hi–C derived TAD boundaries that were co-bound by cohesin complex components (derived from GSE44267)^31^. CTCF motif positions were identified within ENCODE ChIP-seq peaks at target sites using JASPAR. Approximately 200–300 bp of motif flanking genomic sequence were used to identify candidate guide RNA sequences. Guide RNA sequences were scored using previously described rules^32^. The highest scoring gRNA sequences overlapping the CTCF motif were identified and synthesized with vector compatible overhangs (IDT).

### Chromatin Immunoprecipitation (ChIP) assays

ChIP was performed as previously described^33^. Briefly, cells were crosslinked in 1% formaldehyde at 37 °C for 10 min, quenched with glycine, lysed on ice in buffer containing 1% SDS, diluted to a final concentration of 0.3% SDS and sonicated for 5 min total time at ~10 W power. Sonicated chromatin was incubated with the relevant antibody overnight at 4 °C with rotation after dilution to 0.1% SDS. Antibody-bound chromatin was isolated using Protein G magnetic beads (Thermo). Bound complex was washed several times and eluted from beads at 65 °C for 1 h with shaking. Eluted complex was treated with RNase (Roche) for 30 min at 37 °C and proteinase K (Thermo) for 3 h at 63 °C while crosslinks were reversed concurrently. Eluted DNA was isolated using 2× Ampure XP magnetic beads (Beckman) and quantitated using Qubit dsDNA HS (Invitrogen). Antibodies used: CTCF (D31H2), diluted 1:240 (Cell Signaling Technologies, catalog #3418), H3K9me3, diluted 1:750 (abcam, catalog #ab8898).

ChIP-qPCR: Real-time PCR was performed to quantitate ChIP DNA using SYBR Select Master Mix (Applied Biosystems). Primers to quantitate IP DNA in CTCF peaks were generated using the Geneious software program (Biomatters). Each sample was internally normalized using a positive control locus that is constitutively enriched across samples. Enrichments relative to this positive control were compared between experimental and control samples to obtain relative quantitations for each epitope.

Sequencing library preparation: Library preparation was performed as previously described[9] using 5 ng (CTCF and H3K27ac), or 20 ng (H3K9me3) of ChIP DNA input. Paired-end 38 bp reads were generated on a NextSeq500 (Illumina). H3K27ac sequencing reads for HEK293 were obtained from GEO under accession GSM2439222. Reads were uniquely aligned to hg19 using BWA and peak calling was performed using HOMER.

### DNA methylation

Genomic DNA was isolated from 1 × 10^6^ cells using Quick-gDNA (Zymo) following manufacturer’s instructions. Methylation sensitive digestion: Genomic DNA was mass normalized to 100 ng and completely digested using the methylation-sensitive restriction enzyme Hin6I (Thermo) according to manufacturer’s instructions. Mock digested genomic DNA prepared from the same dilution was processed in parallel without addition of enzyme. Quantitation of digested DNA: Digested and mock digested samples were quantitated at target sites by real-time PCR as described above. DNA methylation percentage was calculated as the fraction of undigested genomic DNA retained in the digested samples.

### Chromosome conformation analysis (4C-seq)

4C sample preparation was performed as previously described by Splinter et al. using NlaIII (NEB) and Csp6I (Thermo)^34^. Viewpoint primers were selected from a previously reported database^35^.

Primers sequences were concatenated with Illumina compatible adapter sequences and unique barcodes and synthesized as Ultramer oligos (IDT). Final library amplification was set up on ice using 3.2 µg of purified 4C sample DNA, 2.5 µM primers, and Ultra II Q5 Master Mix (NEB) split into 16 × 50 µl PCR reactions with extension at 65 °C. Amplification products were purified using 1.5× Ampure XP (Beckman) bead purification. Size distributions were established using a Bioanalyzer DNA 12000 Assay (Agilent).

Paired-end sequencing reads were generated as above and analyzed with the 4C-ker pipeline as previously described to identify genomic loci with differential chromosome contacts between conditions^36^.

### Gene expression analysis

RNA extraction: RNA was extracted from 1–2 × 10^6^ cells using the RNeasy kit (Qiagen) following manufacturer’s instructions.

RNA-seq: Sequencing libraries were generated using NEBNext Ultra II RNA Library Prep Kit (NEB) after poly(A) mRNA isolation following manufacturer’s instructions. A minimum of 30 × 10^6^ Paired-end reads were generated on a NextSeq500 (Illumina). Sequencing reads were aligned to hg19 using STAR, transcripts counts were derived using RSEM, followed by analysis with DESeq2 to identify differentially expressed genes. Gene Ontology enrichments of differentially expressed genes was performed using PANTHER^37^.

### Reporting summary

Further information on research design is available in the Nature Research Reporting Summary linked to this article.

## Supplementary information

Supplementary Information

Description of Additional Supplementary Files

Supplementary Data 1

Supplementary Data 2

Supplementary Data 3

Reporting Summary

Source Data

## Data Availability

Data is available in GEO, accession number GSE121998. All other relevant data supporting the key findings of this study are available within the article and its Supplementary Information files or from the corresponding author upon reasonable request. The source data underlying Figs. 1f, 2b, 2d, 2e, 2g and Supplementary Figs. 1e-h, 2a-b, 3a-f are provided as a Source Data file. This study also utilized publicly available datasets GSE44267 and GSM2439222. A reporting summary for this Article is available as a Supplementary Information file.
