# Supplementary material for: Epigenome editing strategies for the functional annotation of CTCF insulators
Source: Nat Commun. 2019 Sep 18;10:4258. doi: 10.1038/s41467-019-12166-w (PMC6751197; doi:10.1038/s41467-019-12166-w)
Supplement: Supplementary file 1 — Supplementary Information [file 41467_2019_12166_MOESM1_ESM.pdf]

Supplementary information - Tarjan et al.

Title: Epigenome editing strategies for the functional annotation of CTCF insulators.

Daniel R. Tarjan, William A. Flavahan, Bradley E. Bernstein

Supplementary Data files:

Supplementary Data 1: gRNA sequences
Supplementary Data 2: ChIP-qPCR primer sequences
Supplementary Data 3: BS-seq primer sequences

Supplementary figures:

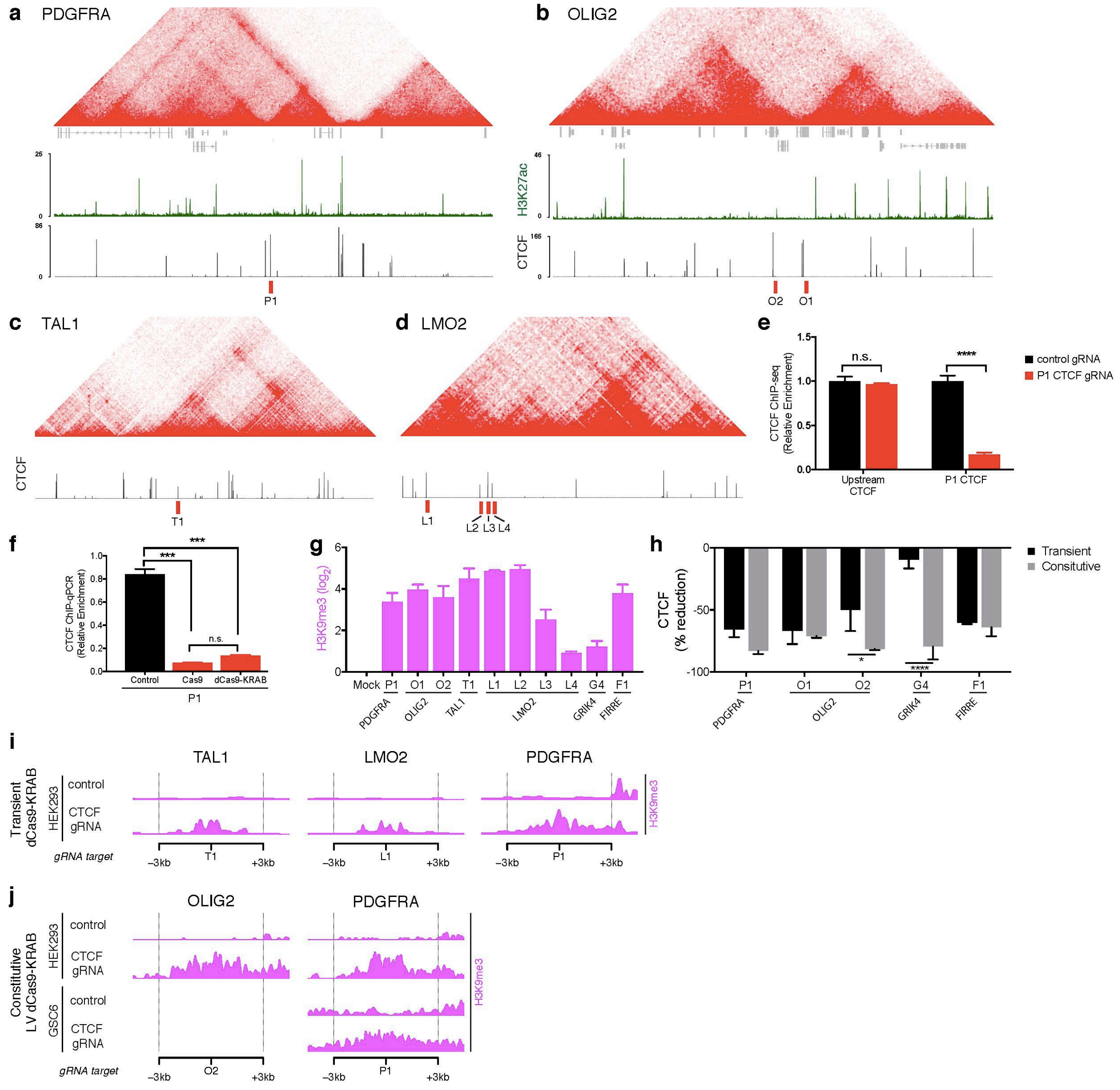

Supplementary Figure 1
S1A-B Genomic views of the PDGFRA and OLIG2 loci show Hi-C contact frequencies from GM12878 (red), RefSeq genes (middle/grey), and ChIP-seq data for H2K27ac (green) and CTCF (grey) from HEK293 cells. Targeted CTCF sites (P1, O2 and O1) are indicated (red boxes).

S1C-D Genomic views of the TAL1 and LMO2 insulator regions show Hi-C data (red) and CTCF ChIP-seq data (gray) as above. Targeted CTCF sites (T1, L1-4) are indicated (red boxes).
S1E Bar plots show change in CTCF occupancy at the P1 CTCF site or its proximal upstream neighbor CTCF site, measured by ChIP-seq in HEK293 cells following transfection with dCas9-KRAB and P1 gRNA, or non-targeting control.
S1F Bar plots show change in CTCF occupancy at the PI site following transfection with dCas9-KRAB, Cas9, or non-targeting control. (See also Figure 1c).
S1G Bar plots show change in H3K9me3 measured by ChIP-qPCR over indicated CTCF sites in HEK293 cells following transient transfection with dCas9-KRAB and indicated gRNAs (see also Fig 1). Data are normalized to non-targeting controls. Error bars, mean ± s.e.m. n ≥ 2.
S1H Bar plots show change in CTCF occupancy measured by ChIP-qPCR in HEK293 cells following epigenome editing by dCas9-KRAB and indicated gRNAs via transient transfection (black bars) or lentiviral transduction (grey bars). Data are normalized to non-targeting controls. Error bars, mean ± s.e.m.
S1I ChIP-seq tracks show H3K9me3 signal at the T1, L1, and P1 insulator sites, in HEK293 cells transiently transfected with dCas9-KRAB plus a single gRNA targeted the respective insulator (bottom row), or a non-targeting control (bottom row). dCas9-KRAB targeting with transient transfection induced a ~3kb domain of H3K9me3 enrichment around the guide RNA target site across all loci tested.
S1J ChIP-seq tracks for H3K9me3 as in (I) for cells constitutively expressing dCas9-KRAB after lentiviral transduction in HEK293 cells (top two rows) and GSC6 cells (bottom two rows, P1 locus only). Constitutive dCas9-KRAB expression induces a H3K9me3 enriched domain that spans 3-6 kb around the guide RNA target site.
Source data for this figure are provided as a Source Data file and in GEO accession GSE121998.

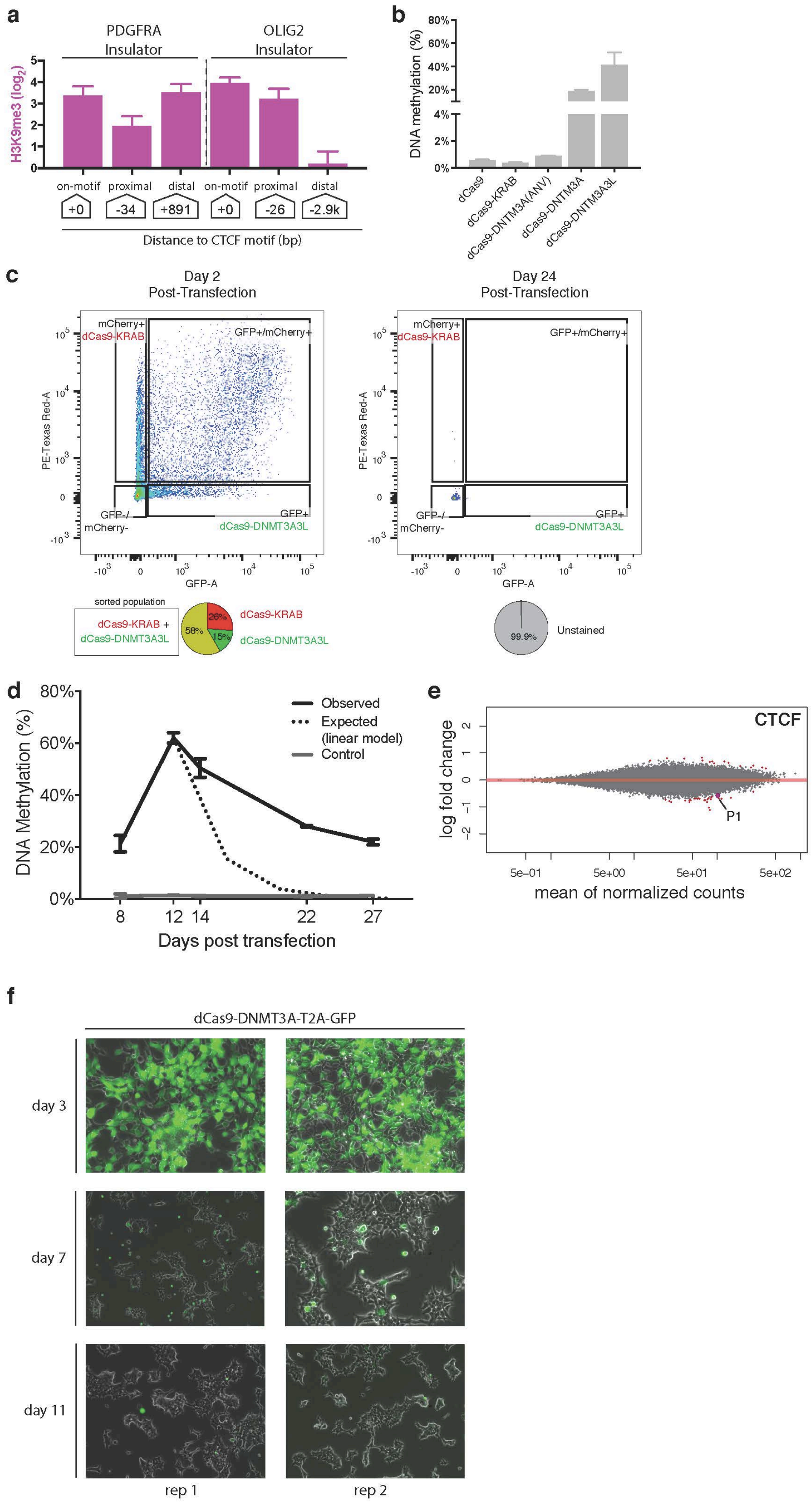

a
PDGFRA Insulator
OLIG2 Insulator
H3K9me3 (log2)
on-motif proximal distal on-motif proximal distal
+0 -34 +891 +0 -26 -2.9k
Distance to CTCF motif (bp)
b
DNA methylation (%)
dCas9
dCas9-KRAB
dCas9-DNTM3A(ANV)
dCas9-DNTM3A
dCas9-DNTM3A3L
c
Day 2 Post-Transfection
Day 24 Post-Transfection
mCherry+ dCas9-KRAB
GFP+/mCherry+
GFP-/ mCherry-
GFP+ dCas9-DNMT3A3L
PE-Texas Red-A
GFP-A
sorted population
dCas9-KRAB + dCas9-DNMT3A3L
26%
58%
15%
dCas9-KRAB
dCas9-DNMT3A3L
99.9%
Unstained
d
DNA Methylation (%)
Observed
Expected (linear model)
Control
Days post transfection
e
CTCF
log fold change
P1
mean of normalized counts
f
dCas9-DNMT3A-T2A-GFP
day 3
day 7
day 11
rep 1
rep 2

Supplementary Figure 2

S2A Bar plots show fold enrichment of H3K9me3 in HEK293 cells measured by ChIP-qPCR following transfection with dCas9-KRAB or dCas9, and gRNA targeted directly to CTCF motifs or to adjacent sequences. White boxes indicate distance of gRNA target sites from CTCF motif. Error bars, mean ± s.e.m. $n \geq 2$. (see also figure 2B).

S2B Bar plot shows DNA methylation at the P1 CTCF motif in HEK293 cells following transient transfection with the constructs listed and single targeting gRNAs. DNA methylation was assayed by methylation sensitive restriction followed by qPCR normalized to mock restricted samples processed in parallel. Error bars mean ± s.e.m. $n \geq 2$. dCas9 and dCas9-KRAB are not expected to have endogenous methyltransferase activity. DNMT3A catalytic domain fusion to dCas9 was assayed in catalytically inactive (DNMT3A(ANV) and active forms (DNMT3A). DNMT3A3L fusion contains an additional DNMT3L multimerization domain.

S2C Plot shows flow cytometry data from HEK293 cells transfected with dCas9-DNMT3A3L-P2A-GFP +/- dCas9-KRAB-P2A-mCherry at day 2 and day 24 post-transfection. Double-positive cells (58%, top-right gate labeled "GFP+/mCherry+") were sorted at day 2 post-transfection (left) and serially passaged as in Fig. 2C. Loss of transiently expressed constructs was verified by flow cytometry of the same samples at day 24 (right). Pie charts (below) summarize proportion of gated cells expressing dCas9-KRAB, dCas9-DNMT3A3L, or both, at day 2 and day 24 post-transfection.

S2D Plots compare observed methylation levels to a theoretical linear dilution model for DNA methylation from peak levels at day 12 through day 27. Observed values are identical to those in Fig. 2G. They reflect methylation levels over the PDGFRA P1 CTCF site in HEK293 cells transiently transfected with dCas9-DNMT3A3L, dCas9-KRAB and P1 gRNA (solid black line). Data for matched mock transfected controls are also shown (solid gray line). The linear dilution model (dashed line) assumes the methylation is not copied during DNA replication and therefore methylation levels are reduced by one-half for each cell division.

S2E Plot shows differential genome-wide CTCF ChIP-seq occupancy in HEK293 cells following transient transfection with both dCas9-DNMT3A3L, dCas9-KRAB and P1 gRNA, compared to mock transfected controls. P1 target insulator site marked (purple).

S2F Time series of fluorescence images show loss of dCas9-DNMT3A fusion expression after transient transfection of HEK293 cells at day 0 followed through day 11.

Source data for this figure are provided as a Source Data file and in GEO accession GSE121998.

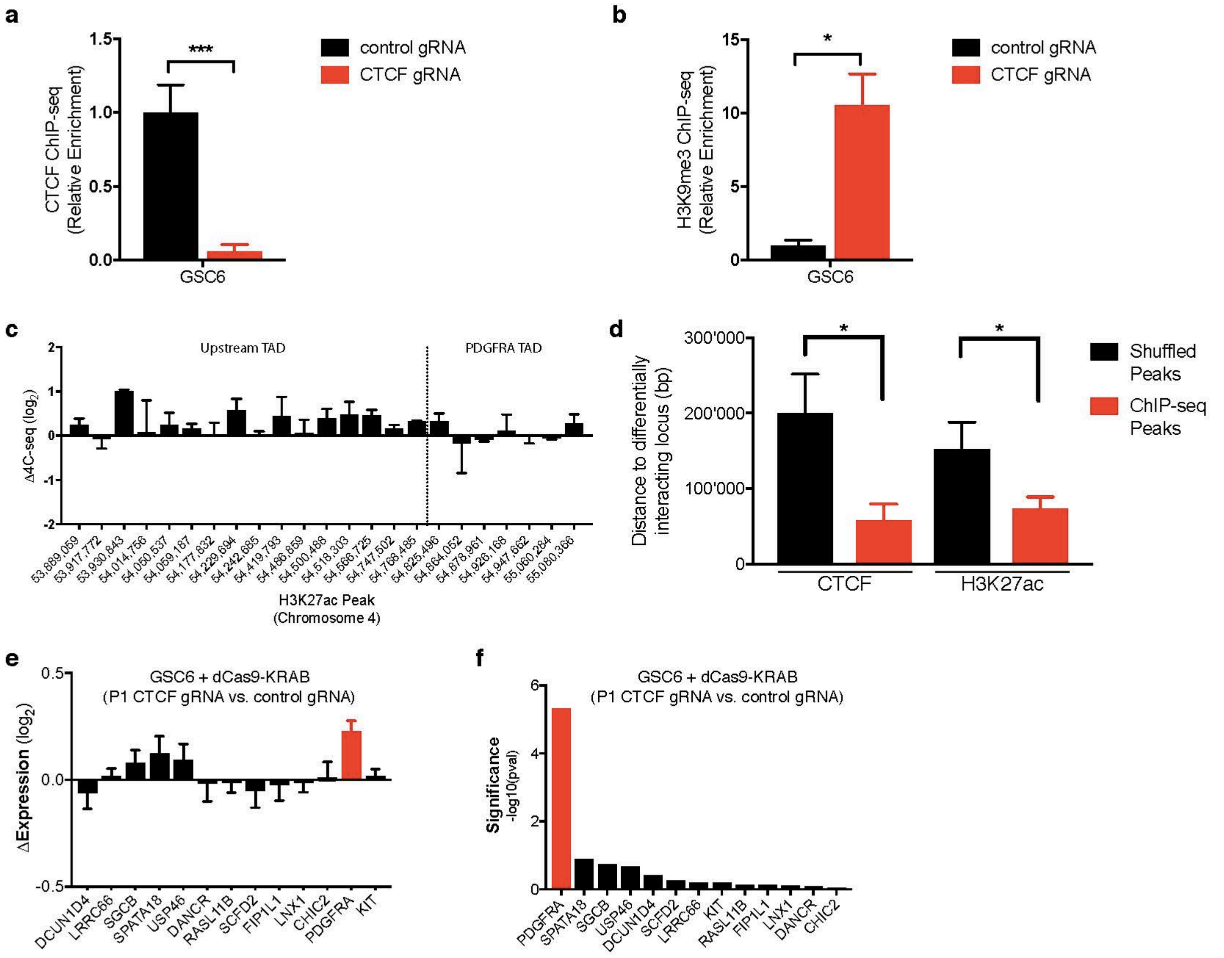

Supplementary Figure 3

S3A Bar plot shows relative CTCF ChIP-seq signal at the P1 CTCF site in GSC6 cells following lentiviral infection with dCas9-KRAB and gRNAs targeting PI (red) or a control sequence (black).

S3B Bar plot shows relative H3K9me3 ChIP-seq signal at the P1 CTCF site for conditions as in S3A.

S3C Bar plot shows quantification of differential 4C-seq interactions between the PDGFRA promoter viewpoint and candidate enhancers (inferred from H3K27ac ChIP-seq peaks) upstream of PDGFRA. Data are shown for GSC6 cells infected with dCas9-KRAB and P1 gRNA, and is normalized to matched non-targeting controls. Dotted line indicates P1 insulator location relative to enhancers sites.

S3D Bar plot shows distances of CTCF and H3K27ac ChIP-seq peaks to loci with differential 4C-seq interactions (red), compared to distances of matched intervals randomly shuffled within the analyzed region (black). These data suggest that the PDGFRA promoter preferentially interacts with CTCF sites and H3K27ac peaks in the neighboring TAD upon insulator disruption.

S3E Bar plot shows differential expression of genes in the TAD upstream of PDGFRA following transfection with dCas9-KRAB and P1 gRNA, relative to non-targeting control.

S3F Bar plot indicated significance of the differential gene expression values in S3E.

Source data for this figure are provided as a Source Data file and in GEO accession GSE121998.

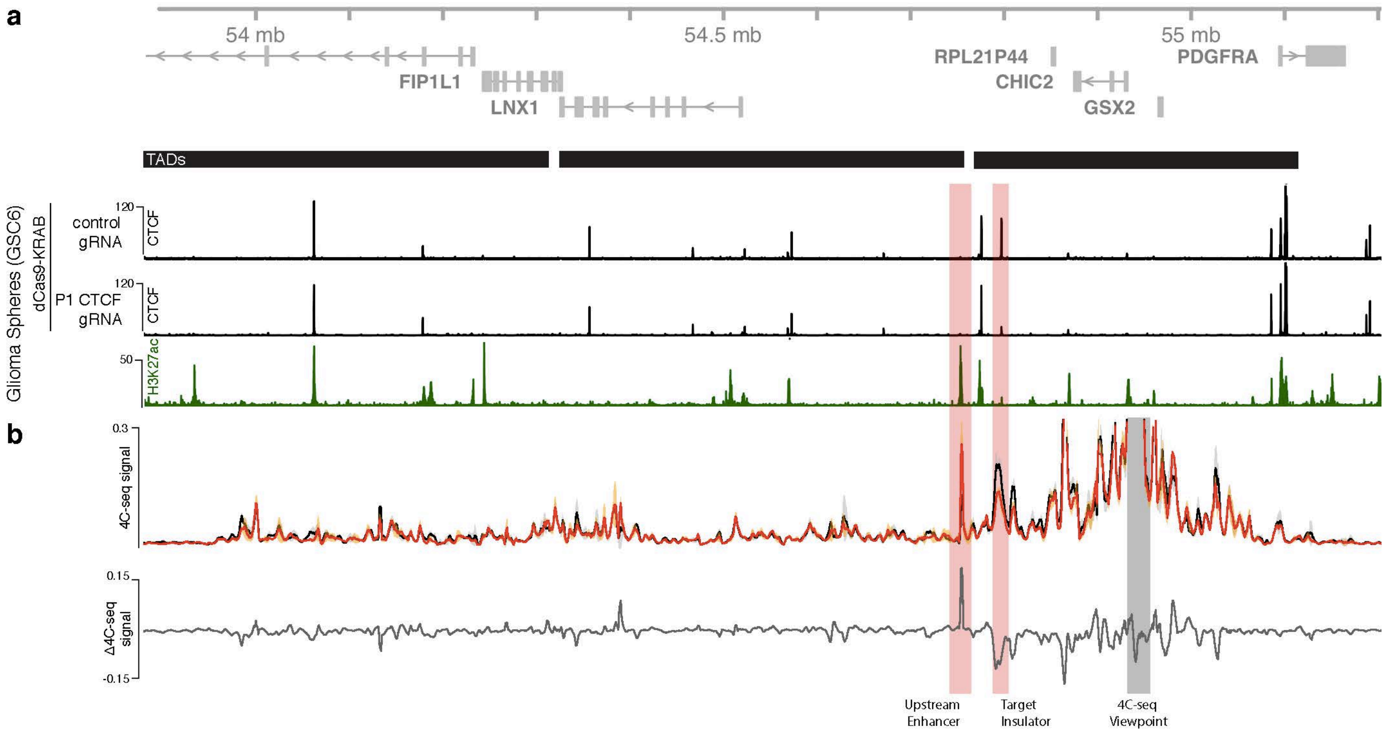

Supplemental Figure 4

S4A As Figure 3A

S4B Plot shows 4C-seq signal measured from a viewpoint near the GSX2 promoter for GSC6 cells transfected with gRNA targeting the P1 CTCF site (red) or non-targeting control (black) as in (a). Signal is shown as the mean across replicates (solid line) ± one standard deviation (shaded area around mean). The difference in 4C-seq signal between the two conditions is shown below the 4C-seq signal (gray line, bottom). Epigenome editing of the P1 site with dCas9-KRAB decreases interactions between the GSX2 promoter and the target insulator, though not to the extent observed for the PDGFRA promoter viewpoint. Interactions also modestly increase with a putative enhancer in the adjacent TAD.

Source data for this figure are provided as a Source Data file and in GEO accession GSE121998.
