# Supplementary material for: Epigenome editing strategies for the functional annotation of CTCF insulators
Source: Nat Commun. 2019 Sep 18;10:4258. doi: 10.1038/s41467-019-12166-w (PMC6751197; doi:10.1038/s41467-019-12166-w)
Supplement: Supplementary file 2 — Description of Additional Supplementary Files [file 41467_2019_12166_MOESM2_ESM.pdf]

Description of Additional Supplementary Files

File Name: Supplementary Data 1

Description: Guide RNA sequences

File Name: Supplementary Data 2

Description: ChIP-qPCR primer sequences

File Name: Supplementary Data 3

Description: BS-seq primer sequences
