# Supplementary material for: Epigenome editing strategies for the functional annotation of CTCF insulators
Source: Nat Commun. 2019 Sep 18;10:4258. doi: 10.1038/s41467-019-12166-w (PMC6751197; doi:10.1038/s41467-019-12166-w)
Supplement: Supplementary file 6 — Reporting Summary [file 41467_2019_12166_MOESM6_ESM.pdf]

nature research

Corresponding author(s): Bradley E. Bernstein

Reporting Summary

Nature Research wishes to improve the reproducibility of the work that we publish. This form provides structure for consistency and transparency in reporting. For further information on Nature Research policies, see Authors & Referees and the Editorial Policy Checklist.

Statistical parameters

When statistical analyses are reported, confirm that the following items are present in the relevant location (e.g. figure legend, table legend, main text, or Methods section).

n/a	Confirmed	
☐	☒	The exact sample size (*n*) for each experimental group/condition, given as a discrete number and unit of measurement
☐	☒	An indication of whether measurements were taken from distinct samples or whether the same sample was measured repeatedly
☐	☒	The statistical test(s) used AND whether they are one- or two-sided<br>*Only common tests should be described solely by name; describe more complex techniques in the Methods section.*
☒	☐	A description of all covariates tested
☒	☐	A description of any assumptions or corrections, such as tests of normality and adjustment for multiple comparisons
☐	☒	A full description of the statistics including central tendency (e.g. means) or other basic estimates (e.g. regression coefficient) AND variation (e.g. standard deviation) or associated estimates of uncertainty (e.g. confidence intervals)
☒	☐	For null hypothesis testing, the test statistic (e.g. *F*, *t*, *r*) with confidence intervals, effect sizes, degrees of freedom and *P* value noted<br>*Give P values as exact values whenever suitable.*
☒	☐	For Bayesian analysis, information on the choice of priors and Markov chain Monte Carlo settings
☒	☐	For hierarchical and complex designs, identification of the appropriate level for tests and full reporting of outcomes
☒	☐	Estimates of effect sizes (e.g. Cohen's *d*, Pearson's *r*), indicating how they were calculated
☐	☒	Clearly defined error bars<br>*State explicitly what error bars represent (e.g. SD, SE, CI)*

Our web collection on statistics for biologists may be useful.

Software and code

Policy information about availability of computer code

Data collection	*Provide a description of all commercial, open source and custom code used to collect the data in this study, specifying the version used OR state that no software was used.*
Data analysis	RNA-seq alignment: STAR v.2.5.1b, RSEM v.1.3.0; ChIP-seq peak calling: HOMER v.4.7, Differential expression analysis: DESeq2

For manuscripts utilizing custom algorithms or software that are central to the research but not yet described in published literature, software must be made available to editors/reviewers upon request. We strongly encourage code deposition in a community repository (e.g. GitHub). See the Nature Research guidelines for submitting code & software for further information.

Data

Policy information about availability of data

All manuscripts must include a data availability statement. This statement should provide the following information, where applicable:

- Accession codes, unique identifiers, or web links for publicly available datasets
- A list of figures that have associated raw data
- A description of any restrictions on data availability

GEO accession number: GSE121998

Field-specific reporting

Please select the best fit for your research. If you are not sure, read the appropriate sections before making your selection.

☒ Life sciences ☐ Behavioural & social sciences ☐ Ecological, evolutionary & environmental sciences

For a reference copy of the document with all sections, see nature.com/authors/policies/ReportingSummary-flat.pdf

Life sciences study design

All studies must disclose on these points even when the disclosure is negative.

Sample size	dCas9-KRAB experiments: 3 biological replicates were used for each condition. dCas9-Dnmt3a3l experiments: 2 biological replicates were used for each condition. Each sample was processed independently and assayed by the relevant method: ChIP-qPCR, ChIP-seq, 4C-seq, RNA-seq.
Data exclusions	No data were excluded from analysis.
Replication	CTCF disruption by the dCas9-effector combinations reported was robust across many genomic loci tested. Each locus tested acts as a replication of the experiment testing CTCF disruption by dCas9-effectors.
Randomization	Covariates including transfection and expression efficiency of the constructs tested were evaluated during early experiments and found to have small impacts on the measured variables. Many reported experiments leveraged flow sorting as described in the main text to further reduce these covariates.
Blinding	Samples were harvested and processed in parallel minimizing the impact of any variable processing between experiments.

Reporting for specific materials, systems and methods

Materials & experimental systems

n/a	Involved in the study	
☒	☐	Unique biological materials
☐	☒	Antibodies
☐	☒	Eukaryotic cell lines
☒	☐	Palaeontology
☒	☐	Animals and other organisms
☒	☐	Human research participants

Methods

n/a	Involved in the study	
☐	☒	ChIP-seq
☐	☒	Flow cytometry
☒	☐	MRI-based neuroimaging

Antibodies

Antibodies used	H3K9me3 (abcam ab8898), CTCF (D31H2) (CST Rabbit mAb #3418)
Validation	H3K9me3 antibody lots were validating by a histone peptide array assay at the Broad Institute Epigenomics Platform.

Eukaryotic cell lines

Policy information about cell lines

Cell line source(s)	HEK293 (ATCC, CRL-1573); GSC6 gliomasphere lines were derived from IDH wild-type tumors resected at Massachusetts General Hospital.
Authentication	HEK293 lines were sourced from ATCC and we rely on their authentication. GSC6 line was sourced from the Massachusetts General Hospital laboratory that generated the line and no further authentication was performed.
Mycoplasma contamination	Cell lines were tested for mycoplasma monthly using a PCR-based detection assay and found to be negative.
Commonly misidentified lines (See ICLAC register)	*Name any commonly misidentified cell lines used in the study and provide a rationale for their use.*

ChIP-seq

Data deposition

☒ Confirm that both raw and final processed data have been deposited in a public database such as GEO.

☒ Confirm that you have deposited or provided access to graph files (e.g. BED files) for the called peaks.

Data access links<br>*May remain private before publication.*	https://www.ncbi.nlm.nih.gov/geo/query/acc.cgi?acc=GSE121998
Files in database submission	For sequencing reads and genome browser tracks please refer to GEO record.<br>ChIP-seq processed peak files included in GEO submission:<br>hek293_ctcf_xpw55-1_fp-stylefactor.bed<br>hek293_ctcf_xpw55-2_fp-stylefactor.bed<br>hek293_ctcf_xpw55-3_fp-stylefactor.bed<br>hek293_ctcf_xpw55-4_fp-stylefactor.bed<br>hek293_ctcf_xpw55-5_fp-stylefactor.bed<br>hek293_ctcf_xpw55-6_fp-stylefactor.bed<br>hek293_h3k9me3_xpw55-1_fp-stylehistone-size1000-minDist2500-F2-L1.5-fdr0.1.bed<br>hek293_h3k9me3_xpw55-2_fp-stylehistone-size1000-minDist2500-F2-L1.5-fdr0.1.bed<br>hek293_h3k9me3_xpw55-3_fp-stylehistone-size1000-minDist2500-F2-L1.5-fdr0.1.bed<br>hek293_h3k9me3_xpw55-4_fp-stylehistone-size1000-minDist2500-F2-L1.5-fdr0.1.bed<br>hek293_h3k9me3_xpw55-5_fp-stylehistone-size1000-minDist2500-F2-L1.5-fdr0.1.bed<br>hek293_h3k9me3_xpw55-6_fp-stylehistone-size1000-minDist2500-F2-L1.5-fdr0.1.bed<br>hek293_ctcf_xpw60-1_fp-stylefactor.bed<br>hek293_ctcf_xpw60-2_fp-stylefactor.bed<br>hek293_ctcf_xpw60-3_fp-stylefactor.bed<br>hek293_ctcf_xpw60-4_fp-stylefactor.bed<br>hek293_ctcf_xpw60-5_fp-stylefactor.bed<br>hek293_ctcf_xpw60-6_fp-stylefactor.bed<br>hek293_h3k9me3_xpw60-1_fp-stylehistone-size1000-minDist2500-F2-L1.5-fdr0.1.bed<br>hek293_h3k9me3_xpw60-2_fp-stylehistone-size1000-minDist2500-F2-L1.5-fdr0.1.bed<br>hek293_h3k9me3_xpw60-3_fp-stylehistone-size1000-minDist2500-F2-L1.5-fdr0.1.bed<br>hek293_h3k9me3_xpw60-4_fp-stylehistone-size1000-minDist2500-F2-L1.5-fdr0.1.bed<br>hek293_h3k9me3_xpw60-5_fp-stylehistone-size1000-minDist2500-F2-L1.5-fdr0.1.bed<br>hek293_h3k9me3_xpw60-6_fp-stylehistone-size1000-minDist2500-F2-L1.5-fdr0.1.bed<br>gsc6ep_ctcf_dCas9_KRAB+NTC_gRNA_rep1_fp-stylefactor.bed<br>gsc6ep_ctcf_dCas9_KRAB+NTC_gRNA_rep2_fp-stylefactor.bed<br>gsc6ep_ctcf_dCas9_KRAB+P1_gRNA_rep1_fp-stylefactor.bed<br>gsc6ep_ctcf_dCas9_KRAB+P1_gRNA_rep2_fp-stylefactor.bed<br>gsc6ep_h3k27ac_dCas9_KRAB+NTC_gRNA_rep1_fp-stylehistone-size1000-minDist2500.bed<br>gsc6ep_h3k27ac_dCas9_KRAB+NTC_gRNA_rep2_fp-stylehistone-size1000-minDist2500.bed<br>gsc6ep_h3k27ac_dCas9_KRAB+P1_gRNA_rep1_fp-stylehistone-size1000-minDist2500.bed<br>gsc6ep_h3k27ac_dCas9_KRAB+P1_gRNA_rep2_fp-stylehistone-size1000-minDist2500.bed<br>gsc6ep_h3k9me3_dCas9_KRAB+NTC_gRNA_rep1_fp-stylehistone-size1000-fdr0.1.bed<br>gsc6ep_h3k9me3_dCas9_KRAB+NTC_gRNA_rep2_fp-stylehistone-size1000-fdr0.1.bed<br>gsc6ep_h3k9me3_dCas9_KRAB+P1_gRNA_rep1_fp-stylehistone-size1000-fdr0.1.bed<br>gsc6ep_h3k9me3_dCas9_KRAB+P1_gRNA_rep2_fp-stylehistone-size1000-fdr0.1.bed<br>gsc6ep_4Cseq_GSX2_TSS_dCas9_KRAB+NTC_gRNA_rep1.bedGraph<br>gsc6ep_4Cseq_GSX2_TSS_dCas9_KRAB+NTC_gRNA_rep2.bedGraph<br>gsc6ep_4Cseq_GSX2_TSS_dCas9_KRAB+P1_gRNA_rep1.bedGraph<br>gsc6ep_4Cseq_GSX2_TSS_dCas9_KRAB+P1_gRNA_rep2.bedGraph<br>gsc6ep_4Cseq_PDGFRA_TSS_dCas9_KRAB+NTC_gRNA_rep1.bedGraph<br>gsc6ep_4Cseq_PDGFRA_TSS_dCas9_KRAB+NTC_gRNA_rep2.bedGraph<br>gsc6ep_4Cseq_PDGFRA_TSS_dCas9_KRAB+P1_gRNA_rep1.bedGraph<br>gsc6ep_4Cseq_PDGFRA_TSS_dCas9_KRAB+P1_gRNA_rep2.bedGraph
Genome browser session<br>(e.g. UCSC)	We will provide this as soon as the record is available.

Methodology

Replicates	dCas9-effector experiments: 3 biological replicates were used for each condition. Each sample was processed independently. Sample belonging to one experiment were processed together in parallel.
Sequencing depth	Please refer to GEO record or SRA pages for most precise sequencing meta-data.
Antibodies	H3K9me3 (abcam ab8898), CTCF (D31H2) (CST Rabbit mAb #3418)
Peak calling parameters	HOMER parameters:<br>H3K9me3 - style: histone; size: 1000; minDist: 2500; F: 2; L: 1.5; fdr: 0.1<br>H3K27ac - style: histone; size: 1000; minDist: 2500<br>CTCF - style: factor

Data quality	*Describe the methods used to ensure data quality in full detail, including how many peaks are at FDR 5% and above 5-fold enrichment.*
Software	ChIP-seq peak calling: HOMER v.4.7

Flow Cytometry

Plots

Confirm that:

☒ The axis labels state the marker and fluorochrome used (e.g. CD4-FITC).

☒ The axis scales are clearly visible. Include numbers along axes only for bottom left plot of group (a 'group' is an analysis of identical markers).

☒ All plots are contour plots with outliers or pseudocolor plots.

☒ A numerical value for number of cells or percentage (with statistics) is provided.

Methodology

Sample preparation	Cells were isolated from the growth medium, washed 3x in PBS, and finally resuspended in PBS+2%FBS + 1mM DAPI for sorting.
Instrument	Cells were sorted on a FACSAria Fusion Cell Sorter in a BSL2+ enclosure.
Software	FCS files were analyzed in FlowJo.
Cell population abundance	Post-sort populations were >99% viable by DAPI stain analysis and >99% positive for the sorted marker.
Gating strategy	Samples were gated to isolate singlet cells and avoid any debris from the cell harvest process. Gates for the target fluorophore (marked in the extended data figures) were determined to allow separation of each population without overlap.

☒ Tick this box to confirm that a figure exemplifying the gating strategy is provided in the Supplementary Information.
